# Territorial Resilience of Metropolitan Regions: A Conceptual Framework, Recognition Methodologies and Planning Response—A Case Study of Wuhan Metropolitan Region

**DOI:** 10.3390/ijerph19041914

**Published:** 2022-02-09

**Authors:** Mengjie Zhang, Chong Peng, Jianfeng Shu, Yingzi Lin

**Affiliations:** School of Architecture and Urban Planning, Huazhong University of Science and Technology, Wuhan 430074, China; zhangmj@hust.edu.cn (M.Z.); jfshu@hust.edu.cn (J.S.); linyingzi@hust.edu.cn (Y.L.)

**Keywords:** territorial resilience, metropolitan regions, territorial planning, Wuhan metropolitan region

## Abstract

As the key link and spatial form of urbanization in China, metropolitan region development has become a strategic frontier issue in the field of regional planning and territorial resilience. This paper defines the essence of territorial resilience of metropolitan regions, analyses the capacity of the system and its elements, and builds a regional planning framework. An evaluation indicator system is constructed to evaluate the territorial resilience level and identify the limiting factors in the Wuhan metropolitan region by utilizing the grey correlation model and the obstacle degree model. The results show that the resilience of Wuhan metropolitan region forms an overall pattern of one core area and four sub-regions in the east, west, north and south. According to the different limiting factors of resilience, cities can be divided into three types: cities limited by both policy and spatial resource factors, cities with lagging socioeconomic factors, and cities with insufficient innovation factors. This paper proposes planning response strategies to enhance resilience from two spatial levels. At the regional level this can be done by building a gradually balanced urban system, establishing three areas based on the degree of resilience factor agglomeration, while at the urban level it can be accomplished by maintaining ecological security, promoting economic agglomeration development and constructing innovation networks.

## 1. Introduction

At present, China’s urbanization process is entering a new stage of pursuing high-quality development, resident living and governance. The CPC Central Committee’s proposal on formulating the 14th five-year plan for national economic and social development and the long-term goals for 2035 highlights important measures to promote new-types of urbanization, such as building resilient cities and modern metropolitan regions. At present, China has a total of 34 metropolitan regions (excluding Hong Kong, Macao and Taiwan), with a total area of about 2.324 million square kilometers, accounting for 24% of the national proportion, and carrying 59% of the national population. In addition, those metropolitan regions are highly dense regions of production. With the in depth development of industrialization, urbanization, marketisation and informatization in China, metropolitan regions have gradually become carriers with high risks. Under the background of spatio-temporal compression, the highly frequent flow of factors in metropolitan regions, the spatial isolation caused by administrative barriers, and the lag in the construction of emergency response have resulted in a series of resilience and safety issues. For example, the Wuhan metropolitan region, as one of the typical metropolitan regions along the Yangtze River Basin, has suffered from frequent floods in recent years, and the outbreak of COVID-19 in Wuhan in 2020 rapidly spread to other areas. The pandemic, coupled with extreme weather and water-related disasters, has had an incalculable negative impact on cities and regions.

The concept of resilience provides a new way to improve the ability of cities and regions to adapt to various risks and changes, and it is gradually becoming a global urban planning and governance action. Territorial planning is the main approach to modernize spatial governance in China, and a key guarantee for urban and regional resilience. By enhancing the capacity of various territorial factors to cope with chronic pressure and sudden disturbances, the territorial system will be better able to cope with, adapt to and recover from multiple risks. As the key link and spatial form of urbanization in China, metropolitan regions have become a strategic frontier issue in the field of regional planning and territorial resilience.

Therefore, facing the challenge of uncertainty and responding to relevant policies, how to achieve resilience development through optimizing the territorial planning of metropolitan regions has become an important topic in urban and rural planning, and also a practical problem to be solved urgently. Two key questions need to be answered: how to define and identify territorial resilience, and how to improve the resilience of metropolitan regions through territorial planning. This paper aims to establish a conceptual framework of territorial resilience and identify the characteristics and key limiting factors of territorial resilience in metropolitan regions. Then, based on the analysis results, some planning strategies and solutions are provided for the improvement of territorial resilience in the Wuhan metropolitan region and other regions, so as to integrate resilience construction into a spatial governance system and effectively consolidate the security foundation for the development of metropolitan regions.

## 2. Literature Review

### 2.1. Theory Origin of Territorial Resilience

Resilience refers to the capability of a system to mitigate shocks, maintain key functions and use resources and opportunities to protect and enhance itself as crises arise [1,2]. When the concept of resilience is combined with cities and regions, it has spatial characteristics and multi-scale spatial forms such as resilient communities, resilient cities and resilient regions. At present, scholars mainly define regional resilience from attributes, processes and capabilities. From the attribute perspective, regional resilience enhancement is essentially the reduction of system vulnerability and the improvement of resource availability [3]. From the perspective of process, most studies focus on the regional resilience dynamic changing process of shock-capability-impact-track-result-new capability [3,4]. From the perspective of capacity, it is generally considered that resilience capacity is the maximum bearing value of the regional system to slow-onset disturbance and sudden shocks, which is mainly composed of resistance, recovery and innovation [3,5].

In China, the concept of territorial resilience first appeared in the ‘Opinions of the CPC Central Committee and the State Council on Establishing and Supervising the Implementation of Territorial Space Planning System’ issued in May 2019. Since then, enhancing territorial resilience has been included as one of the guiding requirements in the compilation guidelines for territorial planning at all levels. At present, theoretical research on territorial resilience is still in its infancy. Foreign research mainly focuses on post-disaster emergency reconstruction planning and vulnerability and multiple risk assessment, prevention and planning methods. The purpose of such research is to explore the operational path of territorial resilience [6]. On the other hand, domestic studies have focused on how the concept of resilience can be integrated into the existing spatial planning and governance system [7,8]. Domestic scholars intend to incorporate resilience and risk management into planning content and discuss technology and decision-making processes to cope with the uncertainty of future social development and various crisis risks [9,10,11,12].

### 2.2. Research on Territorial Resilience Evaluation

Territorial resilience assessment is a basic way to quantify the level of resilience. Some scholars have made beneficial exploration on this, mainly focusing on one-dimensional evaluation, comprehensive evaluation and process evaluation. The first is a one-dimensional evaluation, which mainly focuses on attribute identification, index selection, level evaluation and influencing factor analysis in the fields of regional economy, society, engineering or ecology [13,14,15,16]. The comprehensive evaluation is mainly based on diversified resilience fields and the systematic index system [17,18]. The construction of an index system is mostly based on the connotation of resilience attributes; the required data refer to the three subsystems of resistance, recovery and innovation mentioned above. Attributes assessment usually results in a spatial pattern, analyzes and predicts the spatial distribution characteristics of resilience, and then explores the influencing factors leading to spatial heterogeneity [19].

In terms of evaluation methods, it mainly includes the fuzzy comprehensive evaluation method, the entropy method, principal component analysis, the ranking method for approaching ideal solution (TOPSIS), the analytic hierarchy process method (AHP), analytic network process method (ANP), etc. [20,21,22]. The entropy method is an objective weighting method which determines the index weight according to the information provided by the observed values of each index so as to avoid the deviation caused by subjective factors. However, the entropy weighting method determines the weight of each attribute based on the currently collected information, which depends on the actual problem domain. It is difficult to determine which factors of these indicators are dominant or non- dominant factors, and which factors have strong correlation [23]. The grey correlation analysis model is a method to measure the degree of correlation between factors according to the degree of similarity or difference in the development trend between factors. Its idea is to judge the tightness of the relationship between each data sequence through the similarity of the curve geometry of the sample data column and several comparison data columns [24].

### 2.3. Research on Regional Planning Response

In recent years, resilience adaptation and enhancement policies, strategies and organizational actions have become strategic frontier issues [25,26]. Sharif et al. [27] reviewed the literature focusing on resilience enhancement and discussed seven types of adaptative measures, namely infrastructure, architectural design, urban planning and design, early warning systems and monitoring, policy and management, knowledge and cognition. As one of the public policies, regional planning involves many fields and departments, and has strong legal standing, and is an effective measure to enhance resilience. Regional planning under the guidance of the resilience concept emphasizes the adaptability and convertibility of spatial resource allocation to uncertain disturbances. Accordingly, such regional planning attempts to achieve the optimal operational status of regional development using spatial layouts and land use plans. The existing work of regional planning mainly focuses on the distribution of spatial elements, the optimization of the spatial structure, the organizational mode of the urban system, the coordinated development of cross-administrative areas, and so on [13,14,28]. On this basis, some scholars begin to pay attention to many negative impacts and security issues faced by regional development, and discuss the corresponding planning and control measures qualitatively [29,30,31].

At present, regional security resilience planning has been greatly expanded. The assessment of resource environmental carrying capacity and the suitability assessment of territorial space provide a baseline for regional resilience [32,33]. The comprehensive assessment of security and resilience and the sub-assessment (in economic, social, engineering and ecological fields) guide the development, utilization and protection of territorial space [34,35]. However, there are still some limitations, such as lack of multi-dimensional and multi-field systematic research on resilience and the lack of organic integration among various special plans for resilience.

In terms of research objects, existing studies are more focused on urban agglomerations [36,37,38], and relatively few on metropolitan regions. In fact, metropolitan regions, as an important spatial carrier of China’s new-type urbanization, face some problems in spatial resilience construction, such as the imperfect urban spatial system, unbalanced urban-rural development, the incomplete urban system, the damaged ecological environment [39,40,41], and the insufficiency of elastic space [42]. Therefore, it is urgent to carry out territorial resilience construction in metropolitan regions.

### 2.4. Limitations of Existing Theoretical and Empirical Research

There are three major research gaps in the existing theoretical and empirical research. Firstly, the theory of territorial resilience of metropolitan regions is not mature enough to have a thorough understanding of the essential characteristics of territorial resilience and to construct a systematic research framework based on the uniqueness of Chinese metropolitan regions. Secondly, there are few quantitative studies on the various areas and elements of territorial space in metropolitan regions, let alone the comprehensive quantitative assessment of territorial resilience. This directly restricts the accurate identification of the characteristics and problems of territorial resilience in metropolitan regions, and greatly restricts the further exploration of the promotion mechanisms and modes. Thirdly, there is no mature theoretical system and technology on how to apply the concept of resilience to the planning and governance process of metropolitan regions, and the ability to support the decision making of current territorial planning is also insufficient.

## 3. A Conceptual Framework

### 3.1. Conception of Territorial Resilience of Metropolitan Regions

In this paper, a metropolitan region is defined as a resilient urban system composed of resilient cities. When cities and urban systems face the impact of natural disasters, public health and other emergencies, the territorial resilience capacity system will interact and be unified in the process of resilience stages. From this perspective, the essence of territorial l resilience of metropolitan regions is that territorial planning regulates various elements of the territorial system, such as land use, spatial layout, the ecosystem, urban function, infrastructure and economic industries to cope with shocks. The main function of territorial resilience is to enhance the capacity of metropolitan regions and member cities to identify and resist disasters, reduce disaster losses, and quickly recover from disasters. Ultimately, territorial resilience allows metropolitan regions to prevent potential future risks, and then adapt to and even turn to new development paths.

Based on the above conception, the territorial resilience capacity system is composed of carrying capacity, recovery capacity and innovation capacity, covering nine elements: resource carrying, structural support, environmental maintenance, production run, facility supply, cooperative circulation, technological innovation, public service and social governance.

### 3.2. An Architectural Model of Territorial Resilience of Metropolitan Regions

**Figure 1:**
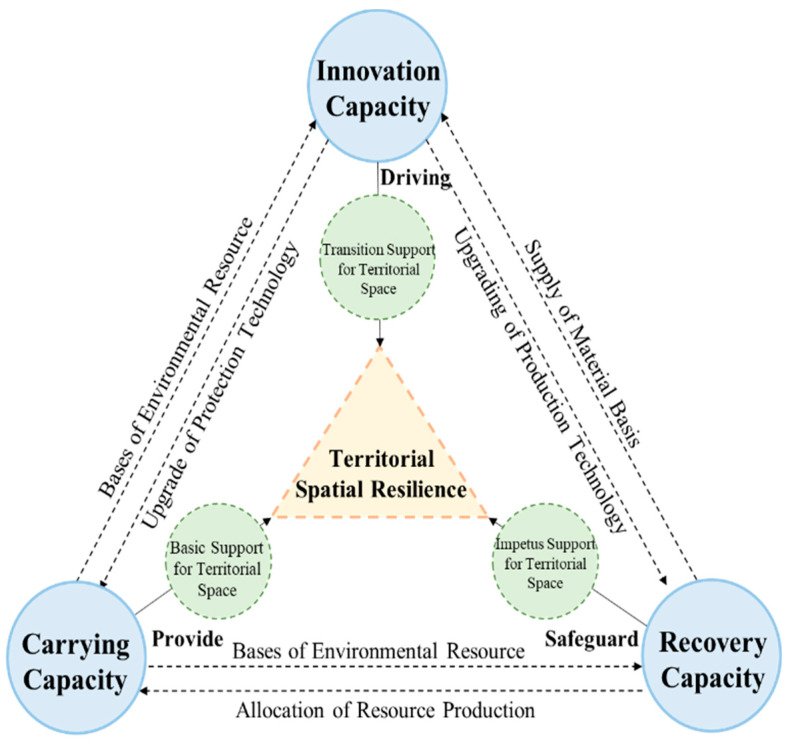
Three-dimensional conjugate angular model of territorial resilience capacity.

According to the above analysis, a three-dimensional conjugation-wrestling model is constructed based on the interaction among carrying capacity, recovery capacity and innovation capacity (Figure 1). Conjugation refers to a stable state in which the elements are closely connected, the overall function is coordinated, and the environment is highly adaptable [43]. Such a state is formed according to certain laws of mutual influence and restriction.

Firstly, carrying capacity means that there are sufficient, stable and safe environmental conditions in territorial space for a certain number of people to carry out activities and to protect themselves from natural disasters, pollution, and other life-threatening health factors. Therefore, the carrying capacity is the foundation of recovery and innovation. Furthermore, the space resources carrying capacity reflects the quantity of land resources endowment and the exploitable scale of lands to human activities. The space structural support capacity determines whether various elements can be reasonably distributed and organized in space. The stronger the ability to maintain the spatial environment, the more effective the use of resources and the stronger the ability to eliminate the negative impact of external events on the spatial environment.

Secondly, recovery capacity is another important component of resilience which involves all aspects of social and economic development. The effect of an economy running in space is the spatial production operation capacity. Under the benign interaction situation, economic factors will combine spatial distribution and aggregation to produce higher outputs and stronger material security under the condition of consuming the same resources. Infrastructure resilience is one of the substantive measures to effectively respond to the crisis [44]. From the perspective of institutional economics, the essence of a city is to provide public goods and services through space. The stronger the supply capacity of space facilities means the more abundant and convenient schools, hospitals, transportation and other facilities. Moreover, with the deepening of the links between cities, the effect of circulation cooperation is constantly enhanced, which has become one of the important factors for the formation of metropolitan regions [45]. The perfection degree, operational efficiency, service level and carrying capacity of circulation space jointly determine whether adjacent cities can form a resilient urban system.

Thirdly, innovation capacity is the key to risk identification, prevention and early warning, as well as the facilitator for raising carrying capacity and recovery capacity to a higher level. Technological innovation is a necessity to accelerate the evolution of resilience stages. The continuous innovation and adoption of new technologies provide the whole society with more options to deal with uncertainties, which fundamentally changes the coping strategies and solutions to crises and subverts people’s lifestyles. Urban space must form a corresponding service capacity. In addition, the introduction of new technologies and new lifestyles will often lead to the reconstruction of the social environment, promote the government to improve governance capacity, adopt new working mechanisms and modes, and respond to the crisis more quickly and efficiently.

### 3.3. The Planning Response Paths for Territorial Resilience Enhancement

**Figure 2:**
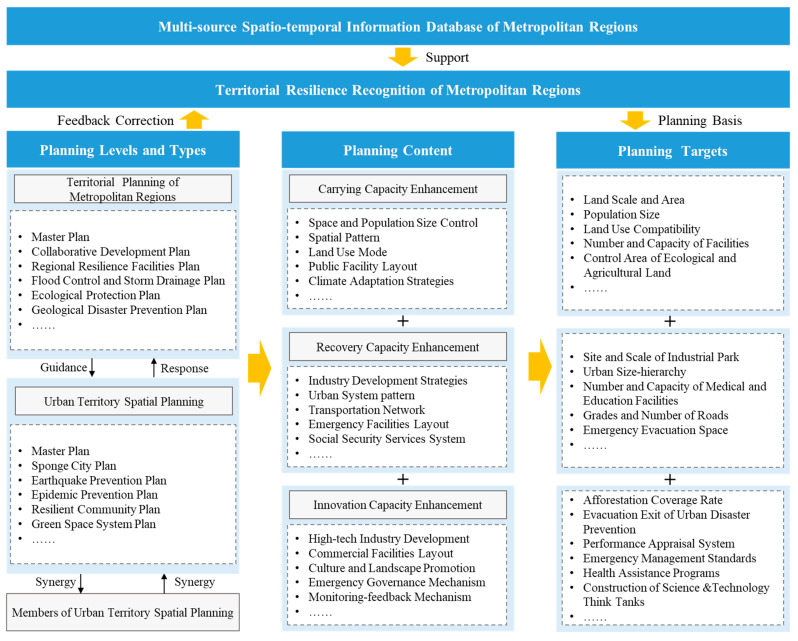
Schematic diagram of metropolitan region planning framework towards territorial resilience enhancement.

The territorial resilience enhancement path of metropolitan regions is not only focused on the spatial planning of a single city, but also a cross-level, multi-direction and multi-domain linkage feedback process between cities and regions. Various types of planning in metropolitan areas and cities improve carrying capacity, recovery capacity and innovation capacity through the implementation of specific planning contents and targets, so as to respond to uncertain challenges such as floods, earthquakes, diseases, economic recession and so on (Figure 2). To be specific, on the one hand, the metropolitan region level determines the overall regional resilience target, allocates and regulates the resilience elements, plans the urban system pattern, and coordinates various resilience plans in different fields. On the other hand, the city level focuses on the implementation of development strategies and governance mechanisms, site and scale, functional division, regional layout, key targets and other aspects determined at the upper level, and also pays attention to the network cooperation among horizontal cities at the same level. A vertical enhancement path for spatial planning highlights the coordination and efficiency of emergency linkage among all levels, so as to ensure that the lower level can respond quickly with the help of the higher level when disasters occur, including facility docking and information communication. In addition, resilience can also be enhanced by a horizontal path through inter-city population flow, industrial cooperation and transportation links, in order to form an integrated network which guarantees territorial security and resilient development.

## 4. Recognition Methodologies

### 4.1. Construction of the Evaluation Indicator System

This paper constructs evaluation indicators following three steps. First of all, the author identifies all optional indicators by referring to existing literature and relevant research [46,47,48,49]. Secondly, the author excludes indicators that are difficult to calculate or without sufficient available data. Thirdly, by adopting a strategy of combining several subjective and objective quantitative methods, such as principal component analysis and expert judgment method, the author ultimately selects effective indicators. The final evaluation indicator system for territorial spatial resilience of metropolitan regions consists of three principal factors, nine elements and 30 specific indicators. Data sources are a combination of dynamic and static data, as well as traditional data and big data. Multivariate data analysis is applied to 15 indicators, accounting for 50% of the total indicators (The statistical data in this paper are from the statistical yearbook of each city in 2019. Big data is retrieved from relevant websites in October 2019; Remote sensing image data are Landsat satellite remote sensing image data with a resolution of 30 m (in 2019)).

The innovativeness of the evaluation indicator system is mainly reflected in the adoption of special indicators that are different from other relevant studies. For example, the calculation of incremental land supply in this study is mainly on the basis of the spatial superposition method, which is based on the spatial distribution map of the administrative region, water area, national ecological reserves, forest area, basic farmland protection area and built-up area of the space unit. The study estimated the stock land supply by idle land area and planned reconstruction project land area. This paper combines remote sensing image data, statistical yearbook data, website data and data from National Development and Reform Commission (The water area and forest area are derived from the calculation of land type identification and classification results of satellite remote sensing data in 2019 based on the GIS platform. The data of basic farmland protection area comes from the Comprehensive Statistical Annual Report of Land and Resources of Hubei Province (2019). This paper obtained the release information of commercial, residential, industrial and storage construction land through the land transfer website by using Octopus software, and calculated the total area of idle land. According to project data in the “13th Five-Year Plan” reserve project database and the “14th Five-year Plan” initial project database of the Hubei Development and Reform Commission, we selected the projects related to the reconstruction and expansion of the current construction land in each spatial unit.) to cope with the difficulty in obtaining land increment and stock data. As for the measurement of spatial morphological structure, this paper identified urban centers by Kernel density analysis through commercial POI data. Then, in terms of evaluating recovery capacity, this paper uses the backstage data of the Fangtianxia (https://www1.fang.com/, accessed on 30 October 2019) and Anjuke (https://wuhan.anjuke.com/, accessed on 30 October 2019) websites so as to measure housing supply through the percentage of housing price changes, and the specific calculation methods are shown in the annex. Furthermore, this paper measures the start-up enterprise vitality indicator and the number of brand stores by using data from Tianyancha (https://www.tianyancha.com/, accessed on 30 October 2019), Dianping (https://www.dianping.com/, accessed on 30 October 2019) and the official Hubei provincial government website (internal website available). Moreover, two indicators, the degree of completion of planning projects and the implementation rate of land supply plan, are selected to reflect the two leading abilities of space governance: planning regulation ability and resource management ability.

Lastly, a variety of geographical and mathematical modelling methods are used to calculate the indicators, and the standardized data matrix is obtained. Furthermore, SPSS19.0 software is used to calculate the weight of each layer by entropy method, as shown in Table 1.

### 4.2. Methods for Evaluating Territorial Resilience

As mentioned in the literature review, the grey correlation model can make up for the defects of the entropy method. In this paper, the grey correlation model based on entropy weight is used to evaluate comprehensive indicators. Firstly, the weight of each indicator is calculated by the entropy weight method, and then the correlation coefficients between indicators are calculated by the grey correlation degree analysis method. The product of the two and the standardized indicator is the final value of that indicator. The specific calculation process is as follows:

(1) The weights of indicators, elements and factors are determined by the entropy weight method.
(2) The grey correlation model method is used to determine the correlation coefficient between indicators, and the indicator value is further calculated based on the weight. The calculation formula is as follows:
  (1) $$Y_{iq}=\sum^{\;}Z_{ij}$$
  where, $Y_{iq}$ is the value of the indicator *q* for the object *i* (*q* = 1,2,…,9). Furthermore, the value of a certain factor is calculated by the following formula:
  (2) $$P_{il}=\overset{3}{\underset{i=1}{\sum }}Y_{iq}\times y_{q}$$
  where $P_{il}$ is the value of the factor *l* for the object *i* (*l* = 1,2,3), and $y_{q}$ is the weight of the indicator $Y_{iq}$. Ultimately, the territorial spatial resilience is calculated by the following formula:
  (3) $$W_{i}=\overset{3}{\underset{i=1}{\sum }}P_{il}\times P_{l}$$
  where $W_{i}$ is the territorial spatial resilience for the object *i*, and $P_{l}$ is the weight of the factor $P_{il}$.

### 4.3. Methods for Identifying Limiting Elements

Based on the resilience level value and the mean value of 39 spatial units in the Wuhan metropolitan region in 2019 (the Wuhan metropolitan region includes Wuhan and the full jurisdiction scope of its surrounding eight cities in a radius of about 100 km (Yellowstone, Xianning, Ezhou, Huanggang, Xiaogan, Xiantao, Tianmen, Qianjiang). The region is further divided into county-level units, including municipal districts (including urban and suburban areas), county-level cities and counties, and provincial directly administered cities, with a total of 39 spatial units.), this paper adopts the method of analysing the limiting factors of regional land use benefit [50], and determines the factors less than the mean value as the weakness of resilience. If it is higher than the mean value, the factor state is 1. If it is lower than the mean value, the factor state is 0. According to the above steps, the following results can be obtained in Table 2. Furthermore, we classified 39 spatial units according to different factor state combinations, obtaining five types of combinations in total (Table 3). On the basis of this, this paper uses the obstacle degree model to clarify the limiting elements inside the lag factor. Specifically, diagnosis is mainly carried out by calculating indicator contribution degree, indicator deviation degree and obstacle degree. Among them, the indicator contribution degree represents the influence degree of a single factor on the whole, that is, the weight $w_{j}$. The indicator deviation degree is the difference between the indicator value of a single element of each spatial unit and the overall level of the metropolitan region, and is set as the difference between the standardised indicator value $x_{ij}$ and 100%. The obstacle degree is the influence value of the single element of each spatial unit on the factor level, and this indicator is the basis for judging limiting factors. Ultimately, the calculation formula is as follows:

(4) $$M_{ij}=\frac{w_{j}\times \left(1-x_{ij}\right)}{\sum_{j=1}^{q}w_{j}\times \left(1-x_{ij}\right)}\times 100\%$$

In the above formula, $M_{ij}$ is the obstacle degree of the indicator *j* for the spatial unit *i*. The larger its value is, the greater the hindrance of this indicator to the improvement of the corresponding factor level of spatial units will be.

## 5. Results and Policy Implications

### 5.1. Characteristics and Problems of Territorial Resilience of Wuhan Metropolitan Region

#### 5.1.1. Characteristics of Resilience Distribution

In this study, the k-medians clustering method is used to systematically cluster the territorial resilience level of the Wuhan metropolitan region. Five tiers are formed from high to low in terms of the resilience level as follows (This paper used the silhouette coefficient (S value) to determine K value. After calculation, the results showed that when K = 3, S = 0.6505; K = 4, S = 0.5882; K = 5, S = 0.7050, which mean five clusters are best.). In the first tier, Wuhan is the only city whose resilience level is much higher than any other cities. The second tier only includes the Huangshi urban area. The third tier includes the Echeng District, Yangxin County, Daye City, Jiangxia District, and the Huangpi District. The fourth tier includes the Xiaogan urban area, Anlu City, Dawu County, Dongxihu District, Huarong District, Liangzihu District, Macheng city, Qichun County, Xiantao City, Tianmen City, Xianning urban area, and Jiayu County. Lastly, the fifth tier consists the remaining 20 districts and counties.

**Figure 3:**
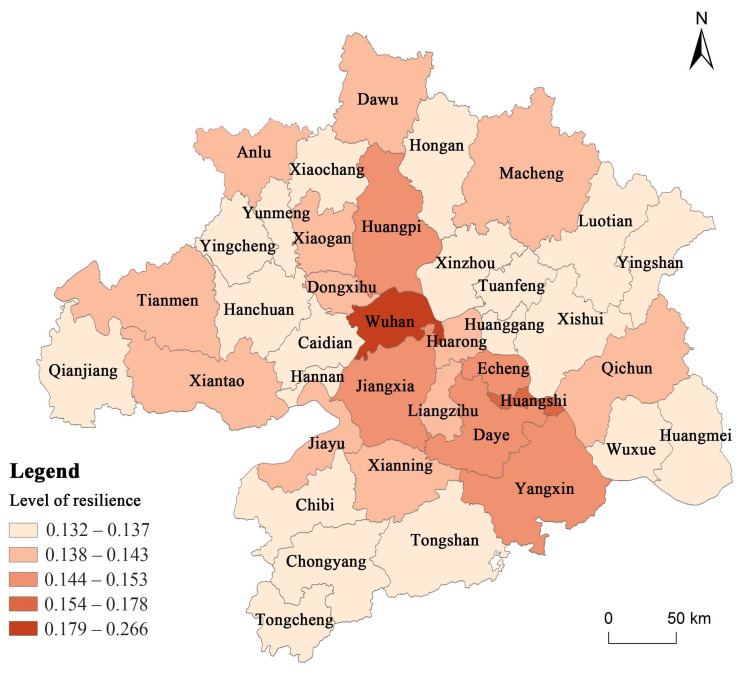
Classification diagram of territorial resilience level of the Wuhan metropolitan region.

**Figure 4:**
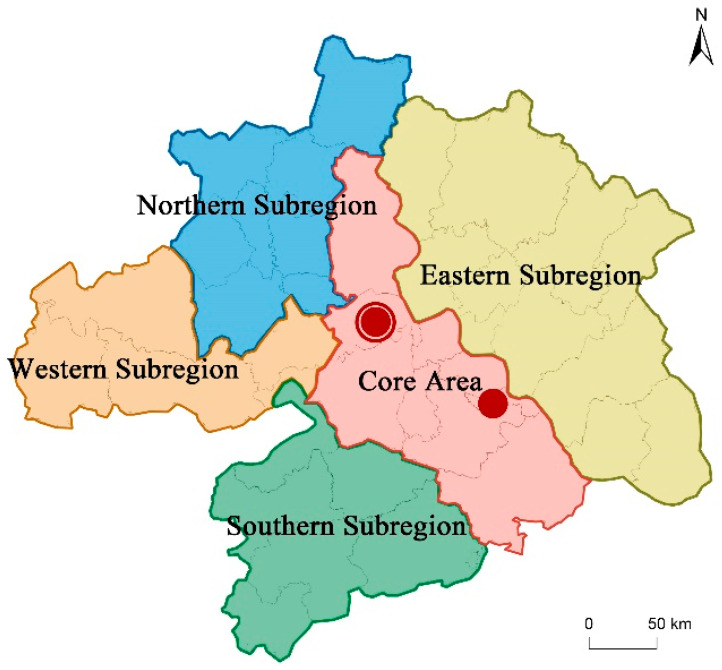
Resilience capacity pattern of Wuhan metropolitan region.

The following facts can be observed after the level of territorial resilience is shown in a hierarchical manner (Figure 3). The municipal district of Wuhan is the absolute core, and its resilience level is much higher than other districts and counties, followed by the sub-center of the Huangshi district, which together constitute a dual core structure. The two core cities have a strong radiating and driving effect on the surrounding areas, forming an approximate “L” shaped horizontal high value interval around the two cores. As the regional circle spreads outwards, the resilience level gradually decreases, forming four sub-regions (Figure 4) in the east, west, north and south, respectively. In each sub-region, there are one or two local spatial units with relatively high resilience levels, but the overall resilience level is not high, presenting a low level of homogeneous distribution. This is consistent with the overall spatial structure pattern of the Wuhan metropolitan region, and local sub-regional clusters are also basically in line with the actual urban clusters, such as the Wu-E-Huang Metropolitan Interlocking Region and the Tian-Xian-Qian Town Cluster. It is implied that although territorial resilience is still subject to the impact of the overall regional development pattern. As a result, the optimisation of the overall regional spatial pattern is the prerequisite to achieve urban resilience development.

#### 5.1.2. Characteristics of Resilience Elements Configuration

In the evaluation, it is found that some cities in the high-value range have an insufficient space resource supply margin, but the social and economic resilience development level is high. This indicates that there is still further development demand and potential. On the contrary, some cities in the low value range have low levels of social and economic development, but sufficient space for improvements. In the actual development of these cities, the land expansion is too fast, and most of the land is used for the development of new towns and industrial parks. The economic and social benefits generated by the new land are not high, which belongs to the extensive development mode and is manifested by the low production efficiency and insufficient public service capacity. All of these are the result of the mismatch between resource allocation and actual development needs. It is difficult for cities to get fundamental adjustment by themselves. Therefore, it is necessary to make unified allocation of resources from the regional level, so as to finally realize the rational distribution of resilience elements.

#### 5.1.3. Characteristics of Resilience Limiting Elements Differentiation

**Figure 5:**
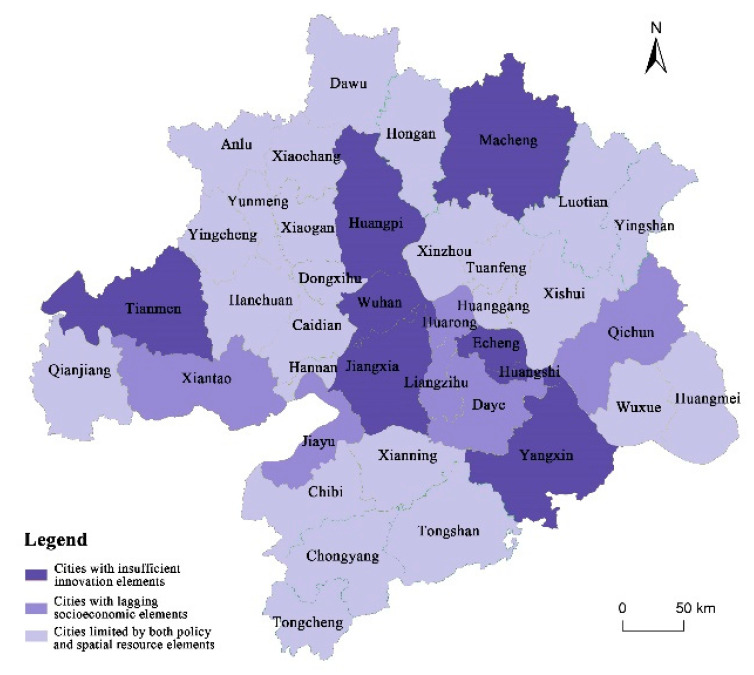
Division of three city types in Wuhan Metropolitan Region.

Through the identification of the limiting factors and elements of the resilience of each spatial unit, this paper can divide cities into three different types: the first type is cities limited by policy and spatial resources, whose biggest limiting elements are carrying capacity of space resources and space governance capacity; the second type is cities with lagging socioeconomic elements, whose are operation capacity of space production and supply capacity of space facilities, which reflects the lack of resilience in social and economic development; the third type is cities with insufficient innovation elements, whose lag factor is innovation capacity (Table 3). The spatial distribution of the three types of cities is shown in Figure 5. It is found that different cities in Wuhan Metropolitan Region have different characteristics, as follows.

- **Cities limited by both policy and spatial resource elements**

**Figure 6:**
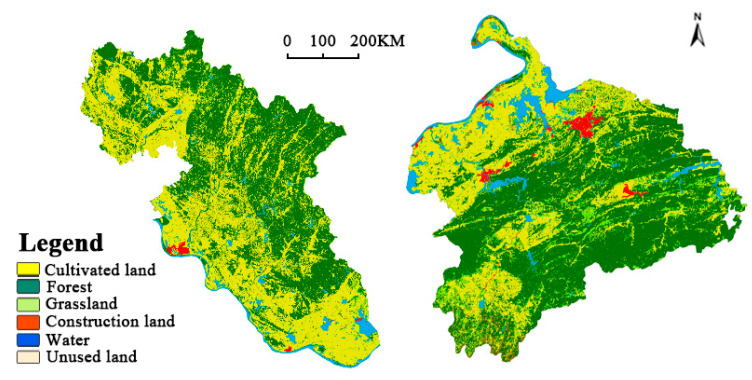
Land use classification map of Huanggang City and Xianning City.

Most of the districts and counties are located in important ecological strategic safety zones, which are restricted development zones and undertake ecological functions such as water conservation, flood regulation and storage, and agricultural production at the national and provincial levels. In the Main Functional Region Planning of Hubei Province, Huanggang belongs to the Dabie Mountain ecological barrier region. From the perspective of maintaining regional ecological security, the policy orientation makes large-scale development and construction activities impossible, and all land use indicators are strictly controlled, setting the upper limit of the carrying capacity of territory to some extent. In addition, from the perspective of the underlying space resources, the landscape pattern inside the city also limits the development, especially in Huanggang city and Xianning city (Figure 6), which are located in Dabie Mountain and Mufu Mountain, respectively. The whole city is occupied and divided by a large number of mountains. In particular, the compactness index and population density are generally low, which shows that the utilization efficiency of spatial resources is low. The advantage lies in the high level of ecosystem services value and environment maintenance quality. The key for such cities to improve territorial resilience is to reorganise and rationally utilise space resources so as to form a harmony with the natural environment.

- **Cities with lagging socioeconomic elements**

These cities’ resource conversion efficiency is low, and they are thus unable to form the corresponding economic industries or social services effect. Compared with the overall level of the region, most social and economic elements are insufficient. A vicious circle from high resource dependence to low industrial efficiency, lack of fiscal revenue, inadequate public service facilities and, finally, to population loss, is formed, and thus those cities face great pressure with regard to urban transformation and upgrading.

Daye, as an example, has been facing the pressure of industrial transformation from an industrial and mining city to a modern industrial city. From the evaluation results, Daye has superior infrastructure construction conditions, which is reflected in the centrality of its transportation network and large number of total freights. The low index of fiscal revenue and employment balance reflects that Daye’s economic strength is too weak to provide sufficient jobs. At the same time, the two negative indicators of construction land consumption intensity and proportion of total investment in fixed assets, are high, indicating that Daye’s economic development mode is still rough and has not reached the target of industrial transformation and upgrading.

Based on the above analysis, it can be found that such cities have a weak potential for population agglomeration. Therefore, on the one hand, it is necessary for them to strengthen the stable working and living environment for residents, and on the other hand, it is necessary to appropriately adjust the scale of space and population development to form a suitable spatial form and functional structure.

- **Cities with insufficient innovation elements**

**Figure 7:**
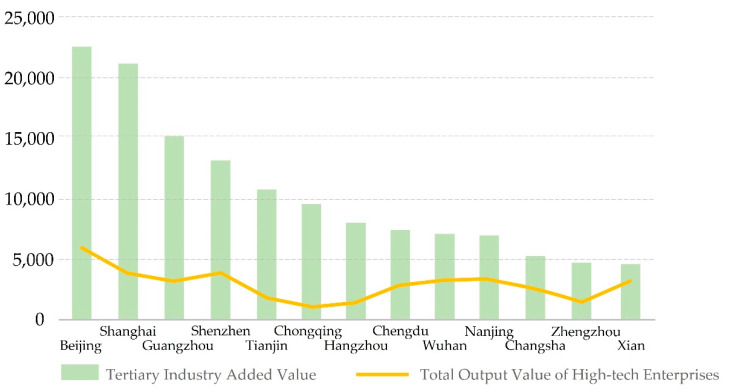
Comparison of innovation level between Wuhan and Major Cities in China.

Wuhan and Ezhou, as typical examples, are cities with a high level of overall resilience, and the level of all elements is relatively balanced. Among them, the spatial service capacity, cooperation capacity of space circulation and operation capacity of space pro-duction are outstanding. Wuhan is the core transportation hub of the central region, and it is also an important commercial center benefitting from superior infrastructure conditions, which is reflected in the large number and scale of business centers as well as the a large quantity of brand stores in the evaluation results. The only element that is relatively weak is innovation capacity. By comparing the added value of the tertiary industry and the total output value of high-tech enterprises in Wuhan with other central cities and the major cities of other countries, it is found that the added value of the tertiary industry in Wuhan is in the middle and lower reaches. Although the value of high-tech industry is in the middle and upper reaches, its advantage is not obvious compared with other cities (Figure 7). Although the number of enterprises and institutions engaged in scientific research in Wuhan is high, the number of invention patents and the ranking in terms of the start-up enterprise vitality index are relatively low, which reflects that Wuhan has not formed an innovation system with close integration of production and research. Therefore, those cities need to strengthen their own innovation capacity first, before improving their spatial correlation with surrounding cities.

### 5.2. Improvement Paths of Territorial Resilience of the Wuhan Metropolitan Region

#### 5.2.1. Building a Polycentric Urban System by a Staged Development Mode

**Figure 8:**
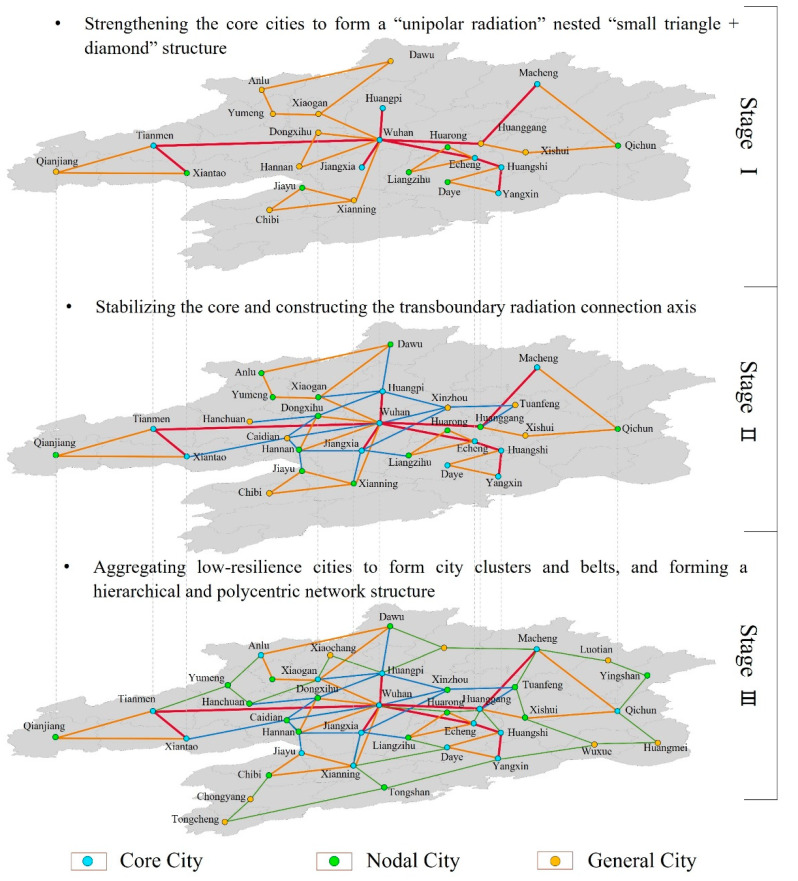
Dynamic spatial structure model of Wuhan metropolitan region.

Based on the hierarchy of the resilient urban system, this paper recommends a network structure consisting of the central city and the peripheral small and medium-sized cities in the metropolitan region, and a dynamic urban organizational system with gradual balance. Cities with insufficient innovation elements have the highest resilience level, and these are known as core cities. Cities with lagging socioeconomic elements have a higher resilience level, and these are known as nodal cities. Cities limited by policy and spatial resources have the lowest resilience level, and these are referred to as general cities. In different development stages, appropriate spatial organization modes should be adopted to make overall arrangements for the direction, sequence, level and mode of development (Figure 8). Specifically, the development process can be divided into the following three stages.

- **Stage I: Strengthening the core cities to form a “unipolar radiation” nested “small triangle + diamond” structure**

This stage should focus on improving the resilience level of the central city itself and the agglomeration, while strengthening the connection between the core city and each node city. With Wuhan as the core, four single-stage radial axes are formed to strengthen the radiation effect of the core and further improve the resilience level of each sub-region. Furthermore, a group of towns with a “small triangle and diamond” structure should be formed around the node cities with high resilience, forming a core area for coordinated development within the sub-region and a connecting area between sub-regions.

- **Stage II: Stabilizing the core and constructing the transboundary radiation connection axis**

In this stage, it is advised to strengthen the connection between the cities in the core area, especially between Wuhan and its surrounding counties, so as to form a solid polygon structure. The peripheral districts and counties of Wuhan are used as a link to connect the cities within each sub-region. In this way, cities form a relatively stable hierarchical structure, and core cities have further strengthened their ability to gather and dominate various elements. In addition, node cities undertake corresponding supporting and supplementary functions. Overall, in this stage, there should form a relatively close network of division of labor and cooperation between cities. However, it is still an incomplete network mode.

- **Stage III: Aggregating low-resilience cities to form city clusters and belts, and forming a hierarchical and polycentric network structure**

At this stage, the resilience level of the lagging cities in the metropolitan region should be improved as soon as possible. It is difficult for these cities to achieve rapid development by themselves, and they must accumulate the potential for development together with surrounding towns. Therefore, each sub-region should take the previously formed “small triangle and diamond” as the basis to drive the development of weak spatial units. When the overall resilience of each sub-region reaches a certain level, it will rely on tangible traffic corridors and invisible element flow to strengthen the connections between cities so as to link the fracture points of the urban network and make up the subsidence areas of the development in the metropolitan region. Finally, a hierarchical and polycentric network is formed.

#### 5.2.2. Forming a Gradient Spatial Distribution Pattern of Elements

**Figure 9:**
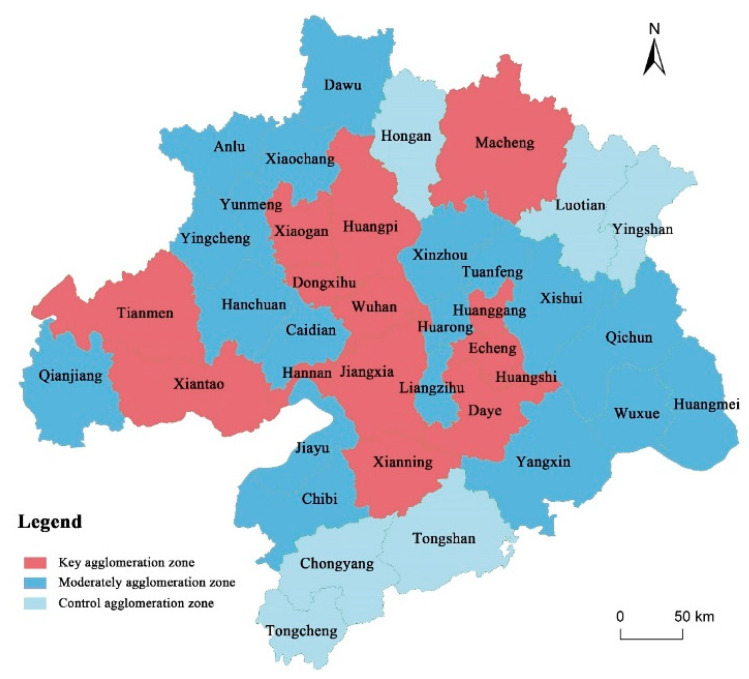
Spatial distribution pattern of elements of Wuhan metropolitan region.

Under the framework of the established regional urban system, areas where elements are highly agglomerated, areas where elements are moderately agglomerated and areas which serve to control elements should be delimited (Figure 9).

**Highly agglomerated area:** Priority should be given to the development of Wuhan and node spatial units in sub-regions. This area has a high level of resilience, great potential for development, and a strong driving effect on the surrounding areas. Due to rapid development, the demand for land is large in some spatial units. However, limited by the natural environment, resource endowment and other factors, the supply of land resources is tight. Therefore, it is necessary to promote the further concentration of land and population by relaxing the land use, optimizing the living environment and improving the comprehensive functions of core cities. In this way, the size of space and population can grow within the limits set by the planning.

**Moderately agglomerated area:** Except for the highly agglomerated areas, county-level spatial units are the main ones in this type of area. Under the background of national policy support, industrial transfer from developed regions and improvement of regional transportation conditions, such areas have certain population gathering capacity by virtue of their advantages in location, natural resources and industrial development conditions. Therefore, priority can be given to supporting districts and counties with prominent comparative advantages to form industrial and functional division and cooperation with highly agglomerated areas, so as to promote moderate growth of space and population.

**Control area:** This area includes Tongshan, Tongcheng and Chongyang in the south, and Hongan, Luotian and Yingshan in the northeast. The main function of such areas is to satisfy regional ecological and agricultural protection requirements. As a result, their territorial development is constrained by both policy and geographical environment. However, these areas also have advantages in agriculture, tourism, leisure and vacation development. Therefore, it is necessary to prevent excessive expansion of space and population, and adopt a characteristic development path fitting local conditions, so as to maintain a balance between population inflow and outflow.

#### 5.2.3. Resilience Enhancement Strategies of Different Types of Cities

- **Strategies targeted at maintaining ecological security**

Most of the cities limited by space resource elements belong to the moderately agglomerated areas and the control areas, so they will not become the cities mainly for carrying population and economic activities in the future. From the perspective of resilience enhancement, cities should focus on the effective integration of spatial resources. Multiple cities should integrate ecological and cultural resources through horizontal linkage to form a specialized tourism network. Therefore, their spatial organization mode should be based on the ecological and natural environment, so as to maintain the forest and farmland ecological open space, which is responsible for material production and safety. The spatial organization mode observes a multi-functional and centralized pattern. Urban functions have more distinctive division of labor that relies on respective resource endowments. For example, cities close to the core city fulfil the roles of industrial undertaking and supporting services, while cities that have abundant natural and cultural resources are suitable for being the elastic buffer space of the metropolitan region.

- **Strategies targeted at promoting economic agglomeration development**

Cities with lagging socioeconomic elements should focus on improving recovery capacity, accelerating industrial transformation, improving production efficiency, and avoiding over-exploitation of resources. From the perspective of spatial distribution, these cities are all located in or close to the core area of the Wuhan metropolitan region, which has locational advantages. Therefore, there is a greater opportunity for them to participate in the regional industrial division. Through regional cooperation, these cities will strengthen their status in the urban system and improve the resilience level. On the one hand, cities should avoid the low efficiency of factor agglomeration caused by the pursuit of “large and comprehensive” industrial development and accurately judge the market demand based on the regional division of labor in combination with their own advantages. On the other hand, cities should implement all-round coordination in the whole process of production organization, including industrial layout, investment attraction, product production, information transmission, logistics transportation and so on.

Spatial organization is guided by the idea of industry-city integration. Taking Daye as an example, the current urban space presents a separation pattern of “urban area, mining area and industrial area”, and the areas are not closely linked. The paper argues that urban areas can be divided into central area, transition area, industrial park, characteristic resource area and ecological protection area. The central area is the key area to improve the service level of the city. The transition area is an area for the integration of industry and city. The characteristic resource area is the key area which demonstrates the unique style of the city.

- **Strategies targeted at constructing innovation networks**

Innovation is the result of a large number of interactions between different participants and institutions. Therefore, the innovation network composed of various innovation nodes and the relationship between them is the key to the development of innovation. The innovation network consists of five innovation nodes: large, medium and small enterprises; universities and scientific research institutions; local governments; financial institutions; and intermediaries. Among them, innovative enterprises are leading innovation activities; universities and scientific research institutions provide knowledge and technology supports; intermediaries and financial institutions are providers of professional services and financial support for innovation networks; local governments play a role in maintaining the innovation network environment.

The materialization of an innovation network requires a certain urban space carrier. The innovation space carrier has developed from the initial industrial park to university science and technology, and then to the knowledge innovation zone of “campus, park and community integration” [51,52], representing the new trend of innovation activity agglomeration. Campuses, parks and communities integrate, interact and even transform each other in the innovation process. Therefore, cities’ spatial layouts mix all kinds of land, blur the boundary of economic, social and cultural space, and increase the resilience of urban space.

## 6. Discussion and Conclusions

This paper defines the essence of territorial resilience of metropolitan regions. Based on the interaction among the carrying capacity, recovery capacity and innovation capacity in the resilience system, an innovative three-dimensional conjugation-wrestling model is created to understand the meaning of territorial resilience. According to the connotation and factor characteristics of territorial resilience, a multi-level and multi-dimensional evaluation indicator system is constructed. This paper selected some innovative indicators in the evaluation dimension, data sources and calculation methods, such as the supply of stock land, the activity of start-up enterprises, and the completion rate of the land supply plan. In order to meet the practical requirements of territorial planning, this paper uses a variety of improved mathematical models and spatial modeling methods to evaluate the territorial resilience level and identify the limiting elements of resilience in the Wuhan metropolitan region.

The main findings are as follows: (1) The territorial resilience of the Wuhan metropolitan region is characterized by central agglomeration and peripheral low-level dispersion, forming an overall pattern of one core area and four sub-regions in the east, west, north and south. (2) There is a mismatch between the supply and demand of resilience elements, and it is necessary to rationalize the spatial distribution of resilience elements at the regional level. (3) According to the different limiting elements of resilience in the Wuhan Metropolitan Region, cities can be divided into three types: cities limited by both policy and space resource elements, cities with lagging socioeconomic elements, and cities with insufficient innovation elements. The difference between them lies in the capacity elements’ state levels and improvement paths.

The current research shows how cities should improve resilience in the context of spatial governance, but does not build a comprehensive and multi-level regional planning framework [53]. When translating adaptation policies into local action plans, it is important to distinguish and integrate the relationship between response paths at the regional level and that at the local or urban level in order to obtain effective governance effects [54]. Overall, building a multi-level regional planning framework and strengthening the horizontal and vertical interaction of regional and urban spatial planning have gradually become the mainstream exploration direction of regional resilience and spatial governance. This paper aims to build a regional planning framework for territorial resilience enhancement, which is not only focused on the spatial planning of a single city, but also a cross-level, multi-direction and multi-domain linkage feedback process between cities and regions, and to propose planning strategies to improve resilience from two spatial levels: regional and urban. At the regional level, it is recommended to build a gradually balanced urban system and adopt appropriate spatial structure patterns at different development stages. In this way, it can strengthen the core cities, stabilize the cores and surrounding structure and form a hierarchical and polycentric network. In addition, this paper believes that three main areas should be established based on the degree of resilience element agglomeration, so as to guide elements to flow to the core area preferentially and facilitate the differentiated development of different areas. At the urban level, this paper puts forward strategies and measures aimed at maintaining ecological security, promoting economic agglomeration development and constructing innovation networks.

The resilience development process includes five stages: risk identification, shock resistance, stress recovery, adaptation and innovation, as well as prevention and early warning [55,56]. At different stages, different factors and elements of resilience show differentiated alternation and interaction characteristics. For example, in the stress recovery stage, the original spatial structure and functions are seriously damaged, and the production operation and circulation cooperation capacity need to be strengthened. With the high accumulation of flow elements and the continuous optimzsation of spatial structure, cities and regions enter a stable state in which enhancement of the control of various factors leads to the rigidity of the overall structure and the lack of adaptability and transformation ability to the drastic changes of the external environment. At this time, it takes innovative activities to restructure and enter the next resilience cycle. Therefore, the focus is to further update and optimise the theories and techniques in exploring the interaction and alternation mechanism of territorial resilience factors in the process of resilience stages so as to ultimately identify change rules and predict trends. There is also a growing awareness of the different risks such as climate change and public health emergencies are having, and they will continue to have spatially specific impacts on urban and regional space [57,58,59,60,61]. The evaluation of territorial resilience of metropolitan regions based on multi-scenario simulation is worth further research. On the basis of the conceptual and evaluation framework constructed in this paper, we can identify the vulnerable areas and linkages of regional networks and predict the dynamic resilience development process under different future disaster scenarios. Putting forward adaptation policies and measures in response to resilience enhancement. Due to the limitation of data, the empirical research object of this paper only includes 39 spatial units in the Wuhan metropolitan region. In the future, the horizontal comparison between cities and regions can be expanded to identify the characteristics and problems of resilience of different metropolitan regions and put forward corresponding planning strategies. In this way, more theoretical and practical results can be produced that need further exploration and improvement in additional research work.

## Figures and Tables

**Table 1:** Factors, elements, specific indicators and weights.

Factors	Elements	Indicators	Attribute	Weight	Data Sources
**Carrying Capacity** **P1** **(0.412432533)**	Carrying Capacity of Space Resources Y1 (0.178724129)	Z1 Incremental Land Supply	+	0.029509685	Calculated from integrated data
		Z2 Stock Land Supply	+	0.130849696	
		Z3 Ecosystem Services Value	+	0.018364748	
	Supporting Capacity of Space Structure Y2 (0.198470348)	Z4 Compactness Index	+	0.017191446	Calculated from remote sensing data
		Z5 Shape Index	−	0.066358282	
		Z6 Number of Commercial Centers	+	0.054869128	Calculated from POI data
		Z7 Population Density of Central Urban Area	+	0.060051492	Calculated from statistical yearbook data
	Maintain Capacity of Space Environment Y3 (0.035238057)	Z8 Days with Air Quality Better Than Grade 2	+	0.020135314	
		Z9 Rate of Centralized Treatment of Urban Sewage	+	0.007050543	
		Z10 Comprehensive Utilization Rate of Industrial Solid Waste	+	0.0080522	
**Recovery Capacity** **P2** **(0.211641716)**	Operation Capacity of Space Production Y4 (0.031445164)	Z11 Construction Land Consumption Intensity	−	0.004883992	
		Z12 Growth Rate of Fiscal Revenue	+	0.011776271	
		Z13 Employment Balance	Section	0.006034287	
		Z14 Proportion of Total Investment in Fixed Assets	−	0.008750614	
	Supply Capacity of Space Facilities Y5 (0.064091519)	Z15 Number of Primary Schools per 10,000 People	+	0.035683676	
		Z16 Coverage of Medical Facilities	+	0.019253617	Calculated from the website data
		Z17 Ratio of House Price Fluctuation	−	0.009154226	
	Cooperation Capacity of Space Circulation Y6 (0.116105033)	Z18 Connectivity of Information Flow	+	0.031106241	
		Z19 Centrality of Transportation Network	+	0.023082372	
		Z20 Total Freight	+	0.06191642	Calculated from statistical yearbook data
**Innovation Capacity** **P3** **(0.375925751)**	Space Innovation Capacity Y7 (0.130817219)	Z21 Start-up Enterprise Vitality Index	+	0.06551139	Calculated from the website data
		Z22 Proportion of High-tech Output in GDP	+	0.015778111	Calculated from statistical yearbook data
		Z23 R&D Investment Intensity	+	0.049527718	
	Space Service Capacity Y8 (0.200424276)	Z24 Proportion of Culture, Education and Entertainment Expenditure in Total Income	+	0.015952741	
		Z25 Number of Scenic Spots above Grade A	+	0.031077873	
		Z26 Afforestation Coverage Rate of Built-up Area	+	0.007065468	
		Z27 Number of Brand Stores	+	0.146328194	Calculated from the website data
	Space Governance Capacity Y9 (0.044684256)	Z28 Planning Project Completion	+	0.017655059	Calculated from the project database data of NDRC
		Z29 Implementation Rate of Land Supply Plan	+	0.015698334	Calculated from integrated data
		Z30 Government Service Satisfaction	+	0.011330863	Calculated from the website data

**Table 2:** Territorial resilience level and factor state of each spatial unit in the Wuhan metropolitan region.

Spatial Units		Carrying Capacity P1	Factor State	Recovery Capacity P2	Factor State	Innovation Capacity P3	Factor State
**Wuhan**	Downtown	0.312856135	1	0.173215505	1	0.267841042	0
	Huanpi	0.186120747	1	0.112490176	1	0.147083737	0
	Xinzhou	0.15694909	0	0.087532234	0	0.141151713	0
	Caidian	0.158707057	0	0.091947508	0	0.151826799	0
	Jiangxia	0.196884647	1	0.099245199	1	0.15114727	0
	Hannan	0.150371773	0	0.098026318	0	0.145210408	0
	Dongxihu	0.154821258	0	0.106689805	0	0.154244415	0
**Huangshi**	Downtown	0.197019264	1	0.117146282	1	0.191134251	0
	Daye	0.183634245	1	0.095507034	0	0.16899491	1
	Yangxin	0.191083016	1	0.114174018	1	0.155397457	0
**Xiaogan**	Downtown	0.156982108	0	0.087424726	0	0.165476031	1
	Xiaochang	0.151243932	0	0.091067818	0	0.152571823	0
	Dawu	0.153426718	1	0.093078001	1	0.178609674	0
	Anlu	0.157067741	0	0.088110648	0	0.19264633	1
	Yunmeng	0.152607759	0	0.085386591	0	0.15577958	1
	Yingcheng	0.150949017	0	0.090138752	0	0.146139577	0
	Hanchuan	0.15281648	0	0.084063831	0	0.149480901	0
**Ezhou**	Downtown	0.208092276	1	0.09689962	1	0.155105645	0
	Huarong	0.197782814	1	0.0848992	0	0.149565546	0
	Liangzihu	0.176738459	1	0.090916581	0	0.148464468	0
**Huanggang**	Downtown	0.150131893	0	0.09265587	0	0.146424357	0
	Tuanfeng	0.148779049	0	0.08981891	0	0.146264403	0
	Hongan	0.150863007	0	0.084918984	0	0.138944429	0
	Luotian	0.162312938	0	0.086635496	0	0.148844919	0
	Yingshan	0.15956004	0	0.101201036	0	0.139426431	0
	Xishui	0.158978669	0	0.095128684	0	0.140864008	0
	Qichun	0.17071931	0	0.085241418	0	0.151901032	0
	Huangmei	0.156093929	0	0.089304221	0	0.147775707	0
	Macheng	0.173588564	1	0.099840027	1	0.153882129	0
	Wuxue	0.155554909	0	0.092063106	0	0.152173264	0
**Xianning**	Downtown	0.161804223	0	0.095181122	0	0.157413475	1
	Jiayu	0.170009611	1	0.092306619	0	0.143075018	0
	Chibi	0.159050793	0	0.095693904	0	0.14462304	0
	Tongcheng	0.155103401	0	0.094583044	0	0.140949498	0
	Chongyang	0.160124322	0	0.091263087	0	0.132814743	0
	Tongshan	0.160719065	0	0.11198916	0	0.1321438	0
Xiantao		0.190375066	1	0.094169832	0	0.145793356	0
Qianjiang		0.162382658	0	0.092973518	0	0.142280555	0
Tianmen		0.170203276	1	0.103063358	1	0.149233539	0

**Table 3:** Different types and factor state combinations of 39 spatial units.

Types	Factor State Combinations	Spatial Units
**Cities limited by both policy and spatial resource elements**	000	Caidian, Luotian, Wuxue, Chibi, Qianjiang, Xiaochang, Huangmei, Xishui, Hanchuan, Tongcheng, Huanggang Dowtown, Xinzhou, Yingcheng, Tuanfeng, Chongyang, Hongan, Dongxihu, Tongshan, Yingshan, Hannan
	001	Anlu, Dawu, Xianning Downtown, Xiaogan Downtown, Yunmeng
**Cities with lagging socioeconomic elements**	100	Huarong, Xiantao, Liangzihu, Qichun, Jiayu
	101	Daye
**Cities with insufficient innovation elements**	110	Jiangxia, Huangpi, Macheng, Tianmen, Wuhan Downtown, Huangshi Downtown, Ezhou Downtown, Yangxin

## Data Availability

Some or all data, models, or code that support the findings of this study are available from the corresponding author upon reasonable request.
